# Solubility and Thermodynamic Analysis of Isotretinoin in Different (DMSO + Water) Mixtures

**DOI:** 10.3390/molecules28207110

**Published:** 2023-10-16

**Authors:** Faiyaz Shakeel, Nazrul Haq, Sultan Alshehri, Miteb Alenazi, Abdulrahman Alwhaibi, Ibrahim A. Alsarra

**Affiliations:** 1 Department of Pharmaceutics, College of Pharmacy, King Saud University, P.O. Box 2457, Riyadh 11451, Saudi Arabia; nhaq@ksu.edu.sa (N.H.); salshehri1@ksu.edu.sa (S.A.); ialsarra@ksu.edu.sa (I.A.A.); 2 Department of Clinical Pharmacy, College of Pharmacy, King Saud University, P.O. Box 2457, Riyadh 11451, Saudi Arabia; mitalanazi@ksu.edu.sa (M.A.); aalwhaibi@ksu.edu.sa (A.A.)

**Keywords:** computational models, isotretinoin, molecular interactions, {DMSO (1) + water (2)} mixtures, solubility, thermodynamic analysis

## Abstract

The solubility and solution thermodynamics of isotretinoin (ITN) (3) in numerous {dimethyl sulfoxide (DMSO) (1) + water (H_2_O) (2)} combinations were studied at 298.2–318.2 K under fixed atmospheric pressure of 101.1 kPa. A shake flask methodology was used to determine ITN solubility, and correlations were made using the “van’t Hoff, Apelblat, Buchowski-Ksiazczak *λh*, Yalkowsky-Roseman, Jouyban-Acree, and Jouyban-Acree-van’t Hoff models”. In mixtures of {(DMSO (1) + H_2_O (2)}, the solubility of ITN in mole fractions was enhanced with the temperature and DMSO mass fraction. The mole fraction solubility of ITN was highest in neat DMSO (1.02 × 10^−1^ at 318.2 K) and lowest in pure H_2_O (3.14 × 10^−7^ at 298.2 K). The output of computational models revealed good relationships between the solubility data from the experiments. The dissolution of ITN was “endothermic and entropy-driven” in all of the {(DMSO (1) + H_2_O (2)} mixtures examined, according to the positive values of measured thermodynamic parameters. Enthalpy was discovered to be the driving force behind ITN solvation in {(DMSO (1) + H_2_O (2)} combinations. ITN-DMSO displayed the highest molecular interactions when compared to ITN-H_2_O. The outcomes of this study suggest that DMSO has a great potential for solubilizing ITN in H_2_O.

## 1. Introduction

**Figure 1:**
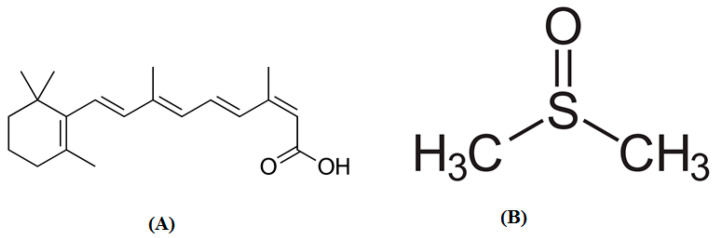
Molecular structures/formulae of (**A**) isotretinoin (ITN) and (**B**) dimethyl sulfoxide (DMSO).

The drug isotretinoin (ITN) is an isomer of retinoic acid, also referred to as 13-*cis*-retenoic acid, that has a *cis* structure [1,2]. Its molecular structure/formula is shown in Figure 1A [3]. ITN was shown to be suitable for the treatment of several malignancies because it plays a significant part in regulating gene expression [4,5]. Additionally, it was discovered to be effective in the treatment of several skin conditions, including psoriasis, skin cancer, and acne [6,7,8,9,10]. It is touted as the most effective treatment for acne [10,11]. A practical approach for the oral administration of ITN in pediatric neuroblastoma patients was recently reported [12] because the patients were unable to swallow marketed tablets or capsules. Due to its poor solubility in water and high lipophilicity, ITN presents challenges in the development of formulations and drug delivery systems especially in terms of liquid dosage forms [13,14]. These challenges in formulation development and drug delivery systems are a poor dissolution rate, poor oral absorption, and poor bioavailability after oral administration [14]. 

Over many years, the pharmaceutical industry has recognized the value of solubility expertise [15,16]. By enabling chemists/scientists to make useful decisions, the solubility data of drugs, particularly in the area of drug development and research, provides useful information to enhance the quality of drug candidates and enhance the success rate clinically [17]. Additionally, the estimation of in vivo pharmacokinetics using solubility data enhances dose prediction [18,19]. The cosolvency technique, one of many that have been researched to enhance the solubility of medications [20,21,22,23], has been widely used in pharmaceutical science and practice [19]. In order to increase the solubility of ITN in this work, the cosolvency technique was applied with dimethyl sulfoxide (DMSO) [Figure 1B], as a cosolvent. The enhancement in ITN solubility using DMSO could resolve several issues of ITN, such as poor aqueous solubility, a poor dissolution rate, poor oral absorption, and poor bioavailability problems. Pharmaceutical drug solubility data are an important physicochemical attribute for a number of industrial processes, including manufacturing, formulation development, and other uses [24,25,26]. The solubilization of ITN in solutions of water (H_2_O) and a cosolvent has not been well reported. To change the physicochemical and basic properties of ITN, a variety of lipid-based formulations, including microemulsions, microemulsion gels, and self-nanoemulsifying formulations, was investigated [27,28,29,30,31,32]. The solubility of ITN in a few environmentally friendly solvents, such as propylene glycol (PG), polyethylene glycol-400 (PEG-400), and carbitol, has been documented at room temperature [28,31]. At temperatures ranging from 298.2 to 318.2 K and an atmospheric pressure of 101.1 kPa, we previously reported the solubility and thermodynamic data of ITN in 11 distinct green solvents, namely H_2_O, methanol, ethanol, 1-butanol, 2-butanol, ethylene glycol, PG, PEG-400, ethyl acetate, carbitol, and DMSO [33].

The stock solution of DMSO has been utilized as a de facto standard for the storage of numerous substances and the distribution of various assays, including solubility assessment [34]. In addition, DMSO is one of the most commonly used cosolvents for solubility enhancement due to its complete miscibility with H_2_O and low chemical reactivity [34,35]. The main limitation of using DMSO is that it affects enzyme activity and cell growth [36]. DMSO is known to influence the protein–ligand binding via solvent viscosity effects and hence it could influence the drug kinetics of the in vivo’s drug disposition [35,37]. It has been reported to reduce ligand–protein binding, which could result in an improved kinetics profile of the drug disposition [35]. The solubility of several weakly soluble pharmaceutical compounds, including raloxifene hydrochloride, sinapic acid, pyridazinone derivatves, baricitinib, meloxicam, and clozapine, has been enhanced using DMSO as a potential solubilizer/cosolvent [26,38,39,40,41,42]. There is no information in the literature regarding the solubility data and thermodynamic parameters of ITN (3) in numerous {DMSO (1) + H_2_O (2)} mixtures at different temperatures (298.2–318.2 K) under constant atmospheric pressure (101.1 kPa). Therefore, this investigation was conducted to determine the solubility and thermodynamic parameters of ITN (3) in numerous {DMSO (1) + H_2_O (2)} mixes, including pure DMSO and H_2_O, at 298.2–318.2 K under atmospheric pressure. The information acquired during the data collection phase of the study may be helpful for the development of dosage forms, pre-formulation studies, and purification of the studied drug.

## 2. Results and Discussion

### 2.1. Solid-State Characterization and Experimental Solubility Data of ITN

The solid-phase characterization of ITN before solubility determination (pure ITN) and after solubility determination (equilibrated ITN) was carried out to investigate the possibility of ITN evolving into polymorphs or solvates/hydrates. The findings of this characterization on pure and equilibrated ITN using differential scanning calorimetry (DSC), powder X-ray diffraction (PXRD), and Fourier transforms infrared spectroscopy (FTIR) investigations are presented in our most recent work [33]. The FTIR, DSC, and PXRD spectra of both samples of ITN were said to be similar and to exhibit similar peak characteristics in our most recent paper [33]. Furthermore, the equilibrated ITN sample did not exhibit any additional FTIR, DSC, or PXRD peaks. According to these results, ITN did not transform into polymorphs or solvates/hydrates. The experimental solubility values of ITN (3) in numerous {DMSO (1) + H_2_O (2)} mixtures at five distinct temperatures and constant pressure are mentioned in Table 1.

**Figure 2:**
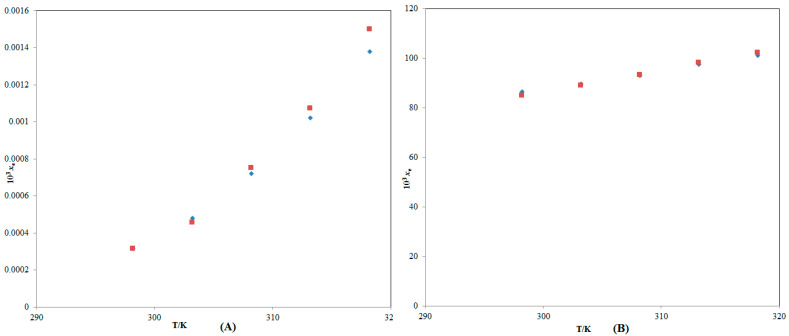
Graphic comparison between ITN mole fraction solubility data (*x*_e_) in (**A**) pure H_2_O and (**B**) pure DMSO with those found in the literature at 298.2–318.2 K. The symbol 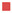 indicates the stated mole fraction solubilities of ITN in (**A**) pure H_2_O and (**B**) pure DMSO, and the symbol 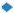 indicates the literature solubilities of ITN in (**A**) pure H_2_O and (**B**) pure DMSO retrieved from reference [33].

**Figure 3:**
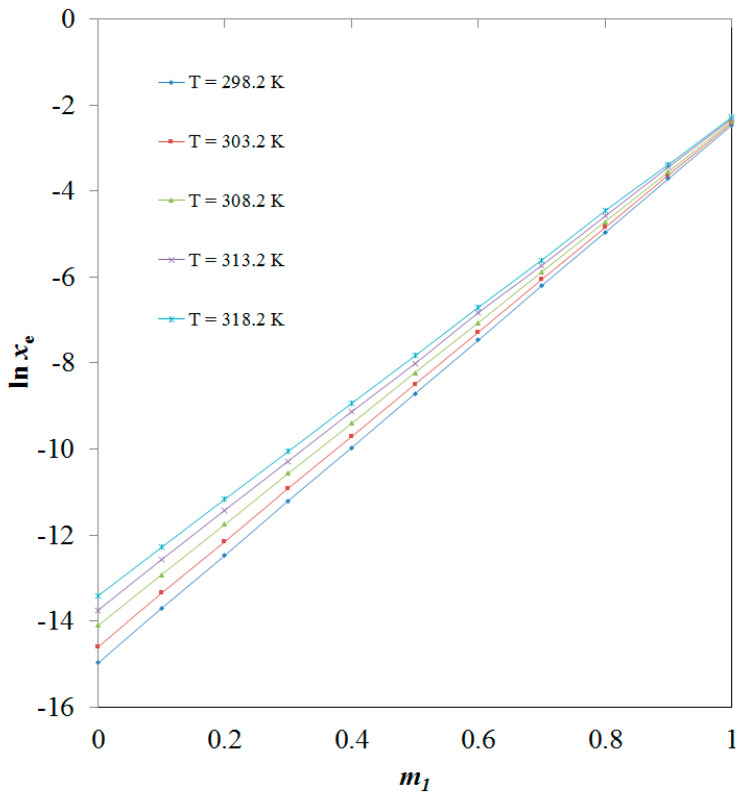
Effect of DMSO mass fraction (*m*_1_) on ITN solubility values (*x*_e_) at 298.2–318.2 K.

ITN solubility (3) in numerous {DMSO (1) + H_2_O (2)} mixes has not been documented. At 298.2–318.2 K, ITN mole fraction solubility data in pure DMSO and H_2_O have been recorded [33]. Figure 2 compares graphically the experimental and literature solubility data of ITN in pure H_2_O and DMSO at 298.2–318.2 K. According to the findings shown in Figure 2, there was a strong correlation between the experimental solubility data of ITN in pure H_2_O and DMSO and those mentioned in the literature [33]. These findings showed that ITN’s experimental solubility statistics agreed well with previously published research [33]. In general, it was found that neat DMSO and neat H_2_O had the highest and lowest mole fraction solubilities of ITN, respectively. The low polarity of DMSO in contrast to the high polarity of H_2_O may be the cause of ITN’s greatest solubility in pure DMSO [38,39,40]. In addition, the enhanced ITN solubility in DMSO could be due to intermolecular interactions between –COOH groups of ITN (Figure 1A) with S=O groups of DMSO (Figure 1B). Temperature and the mass fraction of DMSO both increased the mole fraction solubility of ITN (3) in different {DMSO (1) + H_2_O (2)} solutions. The effect of the DMSO mass fraction on the solubility of ITN in logarithmic mole fractions was also investigated between 298.2 and 318.2 K. Figure 3 provides documentation on the outcomes. At each temperature under study, the ITN solubility increased linearly with the DMSO mass fraction in mixes of {DMSO (1) + H_2_O (2)}. The solubility of ITN in mole fractions increased significantly from neat H_2_O to neat DMSO. Solubilizing ITN in an aqueous media could potentially use DMSO as a solubilizer or cosolvent.

### 2.2. Determination of Hansen Solubility Parameters (HSPs)

ITN’s total HSP (*δ*_t_) was derived using reference [33], and it was found to be 19.30 MPa^1/2^, suggesting low polarity. The HSP values for neat DMSO (*δ*_1_) and neat H_2_O (*δ*_2_) are 23.60 MPa^1/2^ and 47.80 MPa^1/2^, respectively, according to the literature [33]. Calculations revealed that the range of HSP for different {DMSO (1) + H_2_O (2)} mixtures free of ITN (*δ*_mix_) was between 26.02 and 45.38 MPa^1/2^. It was found that the *δ*_mix_ values fell in {DMSO (1) + H_2_O (2)} combinations as the DMSO mass percentage increased. As a result, DMSO mass fraction (*m*) = 0.1 and *m* = 0.9, respectively, were used to obtain the maximum and minimum *δ*_mix_ values. It was discovered, however, that a reduction in *δ*_mix_ values enhanced the solubility values of ITN. ITN (*δ*_t_ = 19.30 MPa^1/2^) and pure DMSO (*δ*_1_ = 23.60 MPa^1/2^) had HSPs that were generally close to one another. The examinations further revealed the greatest solubility of ITN in neat DMSO. Because of this, the ITN solubility data from experiments employing {DMSO (1) + H_2_O (2)} mixes closely mirrored these findings.

### 2.3. Ideal Solubility and Molecular Interactions

Table 1 displays the ideal solubility (*x*^idl^) values for ITN. The calculated values for ITN’s *x*^idl^ range from 4.28 × 10^−2^ to 4.88 × 10^−2^ at 298.2–318.2 K. ITN exhibited substantially higher *x*^idl^ values than experimental solubility (*x*_e_) values in pure H_2_O. At all temperatures examined, ITN’s *x*_e_ values were higher than its *x*^idl^ values in pure DMSO. Because ITN is most soluble in pure DMSO, it can be used as the best cosolvent for ITN solubilization.

Table 2 displays the activity coefficient (*γ*_i_) values for ITN in various {DMSO (1) + H_2_O (2)} combinations at 298.2–318.2 K. At each of the studied temperatures, the ITN’s *γ*_i_ value was at its highest in pure H_2_O. But at each temperature considered, the *γ*_i_ of ITN was lowest in pure DMSO. In comparison to neat H_2_O, the *γ*_i_ values for ITN were substantially lower for neat DMSO. The highest *γ*_i_ for ITN in neat H_2_O may be explained by the least solubility of ITN in H_2_O. These outcomes suggest that, when compared to the ITN–H_2_O combination, the ITN–DMSO combination has the greatest number of solute–solvent interactions at the molecular level.

### 2.4. Correlation of ITN Solubility Data

ITN’s solubility values were correlated by six different computational models, like “van’t Hoff, Apelblat, Buchowski-Ksiazczak *λh*, Yalkowsky-Roseman, Jouyban-Acree, and Jouyban-Acree-van’t Hoff models” [26,43,44,45,46,47,48,49,50,51]. Table 3 displays the findings concerning the correlation with the “van’t Hoff model”. It was determined that this model’s overall root mean square deviation (*RMSD*) was 1.87%. For all cosolvent mixtures as well as neat DMSO and H_2_O, the determination coefficient (*R*^2^) for ITN was calculated to be 0.9940 to 0.9992. Results from the “van’t Hoff model” and ITN (3) experimental solubility values in mixes of {DMSO (1) + H_2_O (2)} showed a strong correlation.

**Figure 4:**
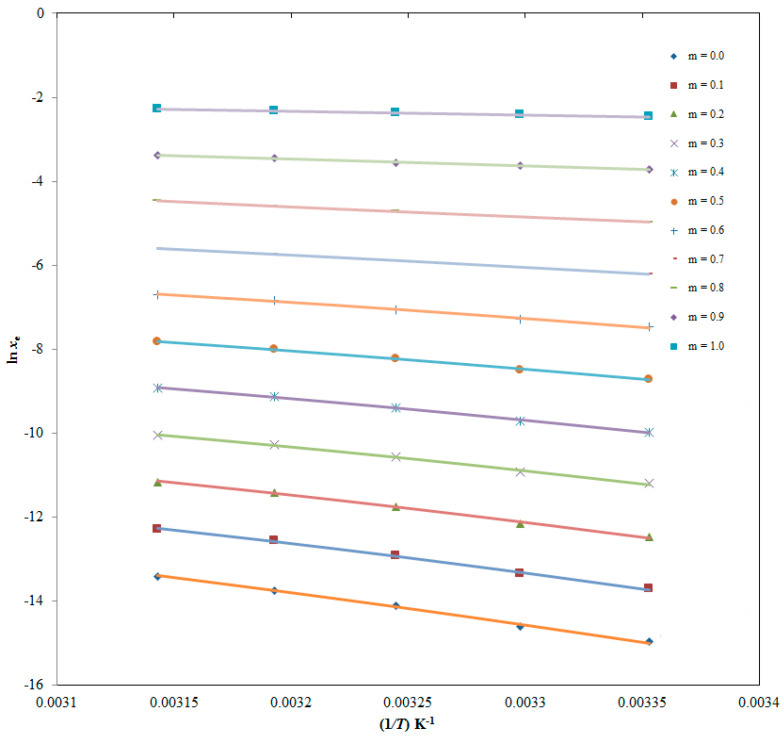
Graphical comparison of experimental ITN (3) solubility data (*x*_e_) with “Apelblat model” in numerous {DMSO (1) + H_2_O (2)} mixes (DMSO mass fraction *m* = 0.0–1.0) against 1/*T*; symbols indicate the experimental ITN solubility data, whereas solid lines indicate the “Apelblat model” ITN solubility data.

In binary solvent mixes, pure H_2_O, and DMSO, experimental and Apelblat solubility data for ITN are graphically correlated in Figure 4. The findings shown in Figure 4 reveal that the experimental solubility values of ITN and the “Apelblat model” correlated well. In Table 4, the Apelblat model parameters and correlation findings for ITN in binary {DMSO (1) + H_2_O (2)} mixes are shown. It was determined that this model’s overall *RMSD* was 1.69%. Including pure DMSO and H_2_O, ITN (3) demonstrated an *R*^2^ of 0.9951–0.9994 in all cosolvent combinations. A significant correlation was also found between the results of the “Apelblat model” and the experimental ITN (3) solubility values in numerous {DMSO (1) H_2_O (2)} mixes.

The findings of the “Buchowski-Ksiazaczak *λh*” correlation for ITN in cosolvent mixtures and neat solvents are shown in Table 5. It was determined that this model’s overall *RMSD* was 3.15%. These results also show a strong agreement between the experimental solubility values from ITN and the “Buchowski-Ksiazaczak *λh* model”.

The results of the correlation with the “Yalkowsky-Roseman model” are shown in Table 6. It was determined that this model’s overall *RMSD* was 2.10%, suggesting a satisfactory connection between the “Yalkowsky-Roseman model” and the solubility data for ITN (3) in various {DMSO (1) + H_2_O (2)} combinations.

In several {DMSO (1) + H_2_O (2)} mixes at varied temperatures and in varied solvent mixes, the solubility value of ITN (3) was likewise correlated to “Jouyban-Acree and Jouyban-Acree-van’t Hoff models” [51]. The results of the correlation with the “Jouyban-Acree and Jouyban-Acree-van’t Hoff models” are shown in Table 7. According to the calculations, the overall *RMSDs* for the “Jouyban-Acree and Jouyban-Acree-van’t Hoff models” are 1.02% and 1.15%, respectively.

### 2.5. Thermodynamic Parameters for ITN Dissolution

**Figure 5:**
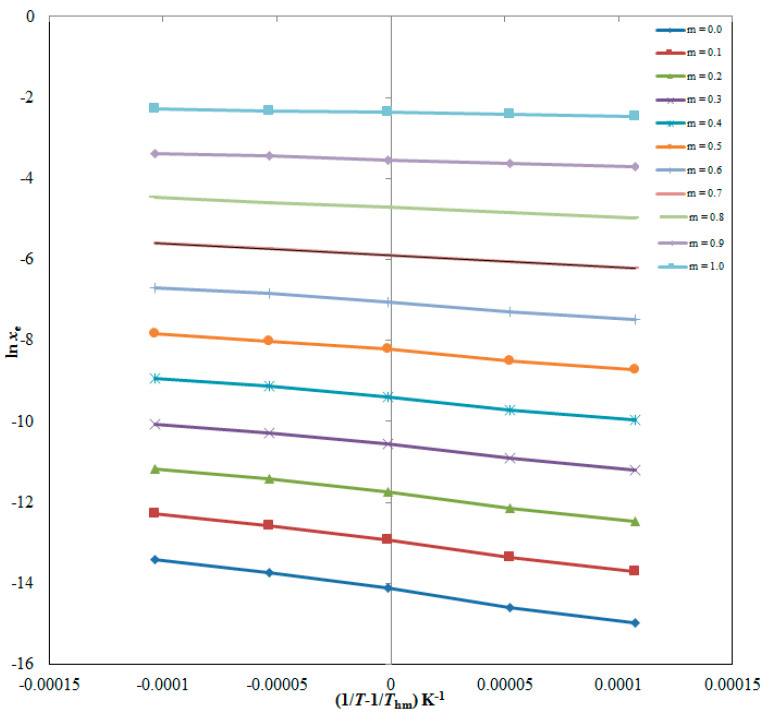
van’t Hoff plots at the mean harmonic temperature (*T*_hm_) for ITN created between logarithmic mole fraction solubility (ln *x*_e_) and 1/*T*-1/*T*_hm_ for ITN in numerous {DMSO (1) + H_2_O (2)} mixtures (DMSO mass fraction *m* = 0.0–1.0).

The van’t Hoff method was used to derive apparent standard enthalpy (Δ_sol_*H°*) values for ITN in all cosolvent mixtures as well as neat DMSO and H_2_O. The linear van’t Hoff graphs of ITN in all cosolvent mixtures, as well as in pure DMSO and H_2_O, are shown in Figure 5 where *R*^2^ > 0.99 was determined, as shown in Table 8. The results for all thermodynamic parameters are likewise shown in Table 8. ITN (3) Δ_sol_*H°* values in numerous {DMSO (1) + H_2_O (2)} mixes and neat DMSO and H_2_O ranged from 7.430 to 63.00 kJ mol^−1^. ITN (3) apparent standard Gibbs energy (Δ_sol_*G°*) values in various {DMSO (1) + H_2_O (2)} mixes and neat DMSO and H_2_O ranged from 6.077 to 36.27 kJ mol^−1^. These results for ITN’s Δ_sol_*H°* and Δ_sol_*G°* revealed “endothermic dissolution” of ITN (3) in various {DMSO (1) + H_2_O (2)} mixes as well as neat DMSO and H_2_O [26,38]. ITN (3) apparent standard entropy (Δ_sol_*S°*) values between 4.392 and 86.78 J mol^−1^ K^−1^ were obtained in numerous {DMSO (1) + H_2_O (2)} mixes as well as in neat DMSO and H_2_O, showing that entropy-driven ITN (3) dissolution occurs in these binary mixtures [26]. In all {DMSO (1) + H_2_O (2)} mixes, including neat DMSO and H_2_O, it was discovered that the dissolution of ITN (3) was “endothermic and entropy-driven” [26,38].

### 2.6. Enthalpy–Entropy Compensation Analysis

**Figure 6:**
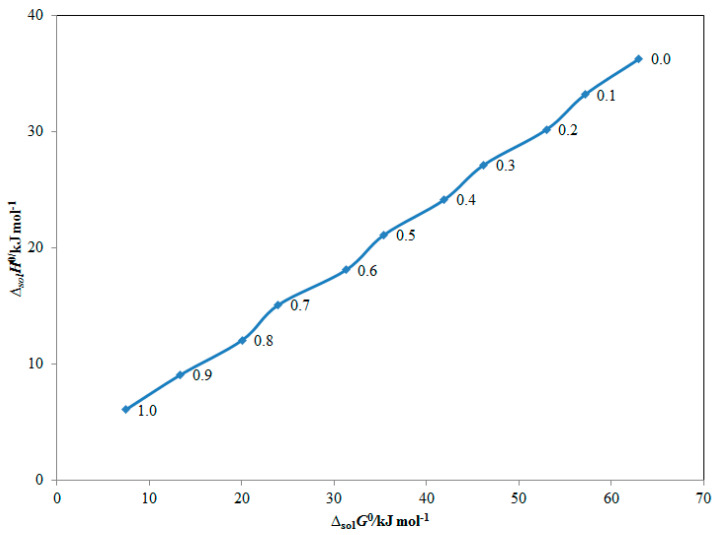
Apparent standard enthalpy (Δ_sol_*H°*) vs. apparent standard Gibbs energy (Δ_sol_*G°*) enthalpy–entropy compensation analysis for solubility of ITN in various {DMSO (1) + H_2_O (2)} mixtures at the mean harmonic temperature (*T*_hm_) = 308 K (DMSO mass fraction *m* = 0.0–1.0).

An enthalpy–entropy compensation analysis was utilized to study the solvation behavior of ITN (3) in various {DMSO (1) + H_2_O (2)} mixes as well as pure DMSO and H_2_O. The results are presented in Figure 6. Figure 6 demonstrates that ITN (3) delivers a linear Δ_sol_*H*° vs. Δ_sol_*G*° curve in all {DMSO (1) + H_2_O (2)} mixtures with neat DMSO and H_2_O, with a slope of larger than 1.0 and R^2^ of greater than 0.99. Based on these findings, it is predicted that the ITN (3) solvation driven mechanism is enthalpy-driven in all {DMSO (1) + H_2_O (2)} mixes as well as in neat DMSO and H_2_O. The fact that ITN solvates more effectively in pure DMSO molecules than in neat H_2_O molecules should be used to explain this mechanism of ITN solvation [26,38]. This led to stronger interactions between ITN-DMSO molecules than ITN-H_2_O molecules. ITN (3) solvated similarly to raloxifene hydrochloride, sinapic acid, pyridazinone derivatives, and baricitinib in numerous {DMSO (1) + H_2_O (2)} mixes as well as in neat DMSO and H_2_O [26,38,39,40].

## 3. Materials and Methods

### 3.1. Materials

ITN was acquired from BOC Sciences (Shirley, NY, USA). DMSO was procured from Sigma Aldrich (St. Louis, MO, USA). Purified H_2_O was procured via a Milli-Q device. The details of each material are summarized in Table 9.

### 3.2. Determination of ITN (3) Solubility in {DMSO (1) + H_2_O (2)} Mixes

Mass measurements of all {DMSO (1) + H_2_O (2)} combinations were taken by a digital analytical balance (Mettler Toledo, Greifensee, Switzerland), which had a sensitivity of 0.10 mg. A series of {DMSO (1) + H_2_O (2)} solutions, with DMSO mass percentages ranging from 0.10 to 0.90, was examined. Three replicates of each {DMSO (1) + H_2_O (2)} combination were taken [26]. ITN’s mole fraction solubility versus mass fraction of DMSO (*m* = 0.0–1.0) and neat DMSO and H_2_O was measured using a shake flask approach at 298.2–318.2 K and 101.1 kPa [52]. In essence, the known amounts of each {DMSO (1) + H_2_O (2)} combination and neat DMSO and H_2_O were combined with extra ITN crystals in triplicate. The equilibrium was achieved by saturating the resultant mixes in a WiseBath^®^ WSB shaking water bath (Model WSB-18/30/-45, Daihan Scientific Co. Ltd., Seoul, Korea) at a shaking speed of 100 rpm for 72 h [33]. In order to evaluate the equilibrium time, the preliminary experiments were performed at different time intervals. It was found that there was negligible change in solubility after 72 h and hence it was selected as the equilibrium time. The saturated solutions were again removed from the shaker after they had reached equilibrium and centrifuged for 30 min at 5000 rpm. A previously established environmentally friendly HPLC method was used to assess the ITN content after the supernatants were isolated and diluted (as required) [53]. The identification of ITN was carried out via a Nucleodur (dimensions: 150 mm × 4.6 mm and particle size: 5 μm) reversed-phase C_18_ analytical column at 298.2 K. The environmentally friendly mobile used was a binary mixture of ethyl acetate and ethanol (50:50% v v^−1^). The mobile phase was delivered with a flow speed of 1 mL min^−1^. The ITN measurements were performed at a wavelength of 354 nm. The sample volume was 20 μL, which was injected using a Waters autosampler. The Analytical GREEnness (AGREE) score was determined to evaluate the eco-friendliness nature of the HPLC method. The AGREE score was predicted to be 0.76 for the present HPLC method, indicating the eco-friendly nature of the HPLC method [53]. ITN mole fraction solubilities (*x*_e_) were calculated using their standard formulae described in our previous work [38,39,40].

### 3.3. HSPs of ITN and Numerous {DMSO (1) + H_2_O (2)} Combinations

A drug’s HSP is directly correlated with how well it dissolves in a neat solvent or cosolvent–H_2_O combination. A medication will reportedly have the highest solubility in a certain solvent when its HSP is close to that solvent’s [54]. As a result, the HSPs for the research medication ITN, neat DMSO, and neat H_2_O were calculated. ITN, neat H_2_O, and neat DMSO *δ*_t_ values were derived from reference [33].

Using Equation (1), the *δ*_mix_ was calculated [55]:

(1) $$\delta_{mix}=\propto \delta_{1}+\left(1-\propto \right)\delta_{2}$$

where *α* is the DMSO volume percentage in the mixture of {DMSO (1) + H_2_O (2)}.

### 3.4. ITN Ideal Solubility and Molecular Interactions

Using Equation (2), we derived the *x*^idl^ of ITN at 298.2–318.2 K [56]:

(2) $$lnx^{idl}=\frac{-∆H_{fus}\left(T_{fus}-T\right)}{RT_{fus}T}+\left(\frac{{∆C}_{p}}{R}\right)[\frac{T_{fus}-T}{T}+\ln\left(\frac{T}{T_{fus}}\right)]\;$$

where *T* is an absolute temperature; *T*_fus_ is the ITN fusion/melting temperature; *R* is a universal gas constant; ∆*H*_fus_ is the ITN fusion enthalpy, and ∆*C*_p_ is the difference in the molar heat capacity of the ITN solid state with its liquid state [57]. Equation (3) was utilized to derive the ∆*C*_p_ for ITN [56,57]:

(3) $$∆C_{p}=\frac{∆H_{fus}}{T_{fus}}$$

The *T*_fus_ and ∆*H*_fus_ values for ITN were taken as 452.7 K and 7.64 kJ mol^−1^, respectively from reference [33]. The ∆*C*_p_ for ITN was calculated to be 16.67 J mol^−1^ K^−1^ using Equation (3). Finally, the *x*^idl^ values for ITN were derived from Equation (2). Equation (4) was used to derive the *γ*_i_ values for ITN in numerous {DMSO (1) + H_2_O (2)} mixes including neat DMSO and H_2_O [56,58]:

(4) $$\gamma_{i}=\frac{x^{idl}}{x_{e}}$$

The chemical basis of molecular interactions between the solute and solvent was explained using ITN *γ*_i_ values.

### 3.5. Correlation of ITN Solubility with Computational Models

The computational verification of experimental drug solubility data is crucial for practical predictions and validations [43,44]. For the correlation of the experimental solubility data of ITN, six distinct computational models, namely “van’t Hoff, Apelblat, Buchowski-Ksiazczak *λh*, Yalkowsky-Roseman, Jouyban-Acree, and Jouyban-Acree-van’t Hoff models”, were utilized [26,43,44,45,46,47,48,49,50,51]. The program used for all modeling tasks was MS Excel 2013. “van’t Hoff model solubility (*x*^van’t^)” of ITN (3) in binary {DMSO (1) + H_2_O (2)} mixtures was derived via Equation (5) [26]:

(5) $$lnx^{van\prime t}=a+\frac{b}{T}$$

where *a* and *b* are Equation (5) model parameters recorded using the least squares methodology [49]. *RMSD* was used to correlate the values *x*_e_ and *x*^van’t^ for the ITN. A formula taken from the literature was used to calculate the *RMSD* [59]. With the help of Equation (6), the “Apelblat model solubility (*x*^Apl^)” of ITN (3) in numerous {DMSO (1) + H_2_O (2)} mixtures was derived [45,46]:

(6) $$lnx^{Apl}=A+\frac{B}{T}+C\ln\left(T\right)$$

where “nonlinear multivariate regression analysis” [59] was used to obtain the Equation (6) model parameters from the experimental ITN solubility values provided in Table 1. In terms of *RMSD*, the results from ITN’s *x*_e_ and *x*^Apl^ were likewise correlated. By Equation (7), the “Buchowski-Ksiazczak *λh* solubility (*x*^λh^)” of ITN (3) in numerous {DMSO (1) + H_2_O (2)} mixtures was derived [47,48]:

(7) $$\ln[1+\frac{\lambda (1-x^{\lambda h})}{x^{\lambda h}}]=\lambda h[\frac{1}{T}-\frac{1}{T_{fus}}]$$

where *λ* and *h* are Equation (7) model parameters.

Because Equations (5)–(7) reflect solubility data at varied temperatures in a certain solvent composition, they cannot be used to forecast the solubility data of a binary solvent combination at varied solvent compositions [51,60,61]. In order to make such forecasts, cosolvency models such as the Yalkowsky–Roseman, Jouyban–Acree, and Jouyban–Acree–van’t Hoff models are needed. With the help of Equation (8), “logarithmic solubility of Yalkowsky-Roseman model (log *x*^Yal^)” for ITN (3) in numerous {DMSO (1) + H_2_O (2)} mixtures was derived [50]:

(8) $$\log x^{\mathrm{Yal}}=w_{1}\log x_{1}+w_{2}\log x_{2}$$

where, *x*_1_ = ITN solubility (3) in DMSO (1); *x*_2_ = ITN solubility in H_2_O (2); *w*_1_ = DMSO mass fraction, and *w*_2_ = H_2_O mass fraction. Drug solubility data in various solvent compositions at a given temperature are linked by Equation (8).

Equation (9) was utilized to derive the solubility of drugs in distinct cosolvent mixtures and temperature ($x_{m,T}$) via the “Jouyban-Acree model” [51]:

(9) $$\ln x_{m,T}=w_{1}\ln x_{1,T}+w_{2}\ln x_{2,T}+(\frac{w_{1}.w_{2}}{T})\sum_{i=0}^{2}J_{i}{\left(w_{1}-w_{2}\right)}^{i}$$

where $x_{1,T}$ and $x_{2,T}$ are the solubility of ITN in DMSO (1) and H_2_O (2) at temperature *T* and *J* terms are Equation (9) model parameters. To calculate the solubility of ITN in cosolvent compositions at the target temperature, the solubility of ITN in neat DMSO and H_2_O must be used as input data. Equations (5) and (9) can be used to create the “Jouyban-Acree-van’t Hoff model” [51] to get around this restriction.

### 3.6. Thermodynamic Parameters

At the mean harmonic temperature (*T*_hm_), all apparent thermodynamic parameters for ITN were determined [56]. The *T*_hm_ was determined using the reported equation [51,56]. The calculated *T*_hm_ for ITN is 308 K. An apparent thermodynamic analysis was applied to derive several thermodynamic parameters. The “van’t Hoff and Gibbs equations” were used to calculate these parameters. The Δ_sol_*H*^0^ values for ITN (3) in various {DMSO (1) + H_2_O (2)} mixtures were calculated using Equation (10) and *T*_hm_ = 308 K [47,62]:

(10) ∂lnxe∂1T−1ThmP=−∆solH0R

The “Δ_sol_*H*^0^” for ITN was derived using the graphed “van’t Hoff” plots between the ln *x*_e_ values of ITN and 1T−1Thm. The van’t Hoff plots for ITN (3) in binary {DMSO (1) + H_2_O (2)} mixes are shown in Figure 5.

Additionally, at *T*_hm_ = 308 K, the Δ_sol_*G*^0^ for ITN (3) in binary {DMSO (1) + H_2_O (2)} mixes was calculated using the Krug et al. methodology via Equation (11) [62]:

(11) $${∆}_{sol}G^{0}=-RT_{hm}\times intercept\;\;\;\;\;\;\;\;\;\;\;\;\;\;\;\;\;\;$$

where, the “van’t Hoff plots” shown in Figure 5 were used to determine the intercept values for ITN (3) in binary mixtures of DMSO (1) and H_2_O (2).

By Equation (12), the Δ_sol_*S*^0^ for ITN (3) in numerous {DMSO (1) + H_2_O (2)} mixtures was derived [56,62,63]:

(12) $${\;\;\;\;\;\;\;\;\;\;\;\;\;\;\;\;\;\;\;\;∆}_{sol}S^{0}=\frac{{∆}_{sol}H^{0}-{∆}_{sol}G^{0}}{T_{hm}}\;\;\;\;\;\;\;\;\;\;\;\;\;\;\;\;\;\;\;\;\;\;\;\;\;\;\;\;\;\;\;\;\;$$

### 3.7. Enthalpy–Entropy Compensation Analysis

An enthalpy–entropy compensation analysis was used, as previously described [26], to evaluate the solvation behavior of ITN (3) in numerous mixes of {DMSO (1) + H_2_O (2)}. Weighted curves of Δ_sol_*H*° vs. Δ_sol_*G*° were generated at *T*_hm_ = 308 K for this experiment [64,65].

## 4. Conclusions

The solubility of ITN in several {DMSO (1) + H_2_O (2)} combinations has not yet been published. This study evaluated the solubility of ITN (3) in binary {DMSO (1) + H_2_O (2)} combinations as well as neat DMSO and H_2_O at various temperatures under constant pressure. In all {DMSO (1) + H_2_O (2)} mixes, including neat DMSO and H_2_O, ITN (3) mole fraction solubilities rose with the temperature and DMSO mass fraction. The maximum and minimum solubilities of ITN in neat DMSO and neat H_2_O, respectively, were found for each temperature studied. Six distinct computational models and experimentally determined ITN (3) solubility data were highly correlated for all {DMSO (1) + H_2_O (2)} mixes, including neat DMSO and H_2_O. It was discovered that all thermodynamic values, including Δ_sol_*H°*, Δ_sol_*G°*, and Δ_sol_*S°*, in numerous {DMSO (1) + H_2_O (2)} mixes as well as pure DMSO and H_2_O were positive, showing “endothermic and entropy-driven” ITN dissolution. Enthalpy drove the ITN solvation process in all {DMSO (1) + H_2_O (2)} combinations as well as in pure DMSO and H_2_O. The collected information from this study may be beneficial for recrystallization, purification, pre-formulation studies, and for the creation of dosage forms for the medicine under study.

## Figures and Tables

**Table 1:** Experimental (*x*_e_) and ideal solubility (*x*^idl^) data of ITN (3) in binary {DMSO (1) + H_2_O (2)} mixes at 298.2–318.2 K and 101.1 kPa ^a^ (values in parentheses are standard deviations).

*m* ^a^	*x*_e_ ^b^				
	*T* = 298.2 K	*T* = 303.2 K	*T* = 308.2 K	*T* = 313.2 K	*T* = 318.2 K
0.0	3.10 (0.07) × 10^−7^	4.60 (0.15) × 10^−7^	7.50 (0.18) × 10^−7^	1.10 (0.02) × 10^−6^	1.50 (0.03) × 10^−6^
0.1	1.12 (0.02) × 10^−6^	1.59 (0.03) × 10^−6^	2.45 (0.05) × 10^−6^	3.47 (0.06) × 10^−6^	4.61 (0.11) × 10^−6^
0.2	3.83 (0.08) × 10^−6^	5.28 (0.17) × 10^−6^	7.87 (0.21) × 10^−6^	1.10 (0.01) × 10^−5^	1.42 (0.02) × 10^−5^
0.3	1.36 (0.01) × 10^−5^	1.82 (0.03) × 10^−5^	2.57 (0.04) × 10^−5^	3.43 (0.06) × 10^−5^	4.29 (0.07) × 10^−5^
0.4	4.68 (0.07) × 10^−5^	6.06 (0.08) × 10^−5^	8.23 (0.10) × 10^−5^	1.08 (0.01) × 10^−4^	1.32 (0.02) × 10^−4^
0.5	1.65 (0.04) × 10^−4^	2.05 (0.05) × 10^−4^	2.69 (0.06) × 10^−4^	3.31 (0.07) × 10^−4^	3.96 (0.08) × 10^−4^
0.6	5.70 (0.10) × 10^−4^	6.88 (0.12) × 10^−4^	8.57 (0.17) × 10^−4^	1.08 (0.01) × 10^−3^	1.22 (0.02) × 10^−3^
0.7	2.02 (0.03) × 10^−3^	2.33 (0.05) × 10^−3^	2.79 (0.06) × 10^−3^	3.24 (0.07) × 10^−3^	3.66 (0.08) × 10^−3^
0.8	6.97 (0.10) × 10^−3^	7.81 (0.12) × 10^−3^	8.96 (0.20) × 10^−3^	1.02 (0.01) × 10^−2^	1.15 (0.01) × 10^−2^
0.9	2.44 (0.02) × 10^−2^	2.66 (0.03) × 10^−2^	2.86 (0.03) × 10^−2^	3.19 (0.04) × 10^−2^	3.39 (0.05) × 10^−2^
1.0	8.47 (0.10) × 10^−2^	8.88 (0.11) × 10^−2^	9.31 (0.12) × 10^−2^	9.80 (0.13) × 10^−2^	1.02 (0.01) × 10^−1^
*x* ^idl^	4.28 (0.03) × 10^−2^	4.43 (0.04) × 10^−2^	4.58 (0.05) × 10^−2^	4.73 (0.06) × 10^−2^	4.88 (0.07) × 10^−2^

^a^ The uncertainties *u* are *u*(*T*) = 0.18 K, *u*(*m*) = 0.0007, and *u*(*p*) = 2 kPa. ^b^ The relative uncertainty *u*_r_ in solubility is *u*_r_(*x*_e_) = 0.04.

**Table 2:** Activity coefficients (*γ_i_*) of ITN in numerous {DMSO (1) + H_2_O (2)} combinations at 298.2–318.2 K.

*m*	*γ*_i_				
	*T* = 298.2 K	*T* = 303.2 K	*T* = 308.2 K	*T* = 313.2 K	*T* = 318.2 K
0.0	1,366,467	972,627.0	611,320.0	441,076.0	326,128.0
0.1	384,000.0	278,000.0	187,000.0	136,000.0	106,000.0
0.2	111,979.0	83,952.80	58,249.00	43,099.10	34,943.20
0.3	31,441.80	24,298.30	17,811.50	13,802.30	11,402.70
0.4	9161.740	7310.360	5568.540	4372.740	3713.1570
0.5	2602.340	2166.250	1703.980	1429.250	1233.060
0.6	751.8650	644.3650	534.7140	438.6560	399.5110
0.7	211.8050	189.9250	164.4500	146.1890	133.4190
0.8	61.48640	56.71900	51.11100	46.46460	42.45130
0.9	17.59830	16.68020	16.02050	14.83990	14.41810
1.0	5.057310	4.988350	4.918850	4.828590	4.790800

**Table 3:** Outcomes for the “van’t Hoff model” in terms of model parameters (*a* and *b*), *R*^2^, and *RMSD* for ITN (3) in numerous {DMSO (1) + H_2_O (2)} mixtures (values in parentheses are standard deviations of model parameters).

*m*	*a*	*b*	*R* ^2^	Overall *RMSD* (%)
0.0	10.402 (0.34)	−7568.3 (512.12)	0.9966	
0.1	9.3196 (0.32)	−6865.0 (432.41)	0.9971	
0.2	8.8695 (0.30)	−6364.6 (402.15)	0.9967	
0.3	7.4104 (0.27)	−5549.1 (321.27)	0.9969	
0.4	6.9041 (0.22)	−5031.7 (301.43)	0.9968	
0.5	5.5507 (0.17)	−4252.5 (284.81)	0.9967	1.87
0.6	5.1279 (0.16)	−3757.5 (253.58)	0.9940	
0.7	3.4432 (0.10)	−2877.3 (194.29)	0.9978	
0.8	3.1037 (0.09)	−2408.9 (180.21)	0.9988	
0.9	1.6606 (0.05)	−1603.6 (64.29)	0.9948	
1.0	0.52410 (0.01)	−892.54 (28.41)	0.9992	

**Table 4:** Outcomes of the “Apelblat model” in terms of model parameters (*A*, *B*, and *C*), *R*^2^, and *RMSD* for ITN (3) in numerous {DMSO (1) + H_2_O (2)} mixes (values in parentheses are standard deviations of model coefficients).

*m*	*A*	*B*	*C*	*R* ^2^	Overall *RMSD* (%)
0.0	454.09 (31.12)	−27954 (182.26)	−65.877 (6.71)	0.9969	
0.1	563.01 (34.58)	−32298 (203.31)	−82.214 (8.45)	0.9978	
0.2	512.95 (32.81)	−29519 (188.16)	−74.847 (7.71)	0.9974	
0.3	521.99 (33.10)	−29183 (183.24)	−76.408 (7.93)	0.9979	
0.4	436.22 (29.82)	−24751 (174.34)	−63.746 (6.94)	0.9977	
0.5	344.90 (26.24)	−19840 (121.63)	−50.388 (5.13)	0.9974	1.69
0.6	347.63 (26.31)	−19489 (119.06)	−50.857 (5.16)	0.9951	
0.7	126.23 (6.13)	−8521.8 (64.31)	−18.230 (1.57)	0.9980	
0.8	−148.36 (7.21)	4553.4 (44.12)	22.479 (1.77)	0.9994	
0.9	44.494 (2.84)	−3574.5 (38.84)	−6.3581 (0.76)	0.9950	
1.0	11.915 (1.01)	−1418.1 (22.89)	−1.6901 (0.10)	0.9992	

**Table 5:** Outcomes of “Buchowski-Ksiazaczak *λh* model” for ITN (3) in numerous {DMSO (1) + H_2_O (2)} mixes (values in parentheses are standard deviations of model parameters).

*m*	*λ*	*h*	Overall *RMSD* (%)
0.0	5.3156 (0.86)	1423.8 (31.23)	
0.1	4.8451 (0.83)	1416.9 (30.62)	
0.2	4.1898 (0.75)	1519.0 (33.18)	
0.3	3.8473 (0.66)	1442.3 (32.51)	
0.4	3.2108 (0.51)	1567.0 (34.21)	
0.5	2.8430 (0.42)	1495.8 (32.91)	3.15
0.6	2.1723 (0.27)	1729.7 (38.44)	
0.7	1.9127 (0.12)	1504.3 (33.61)	
0.8	1.2176 (0.10)	1978.4 (41.18)	
0.9	0.88170 (0.01)	1818.7 (39.42)	
1.0	0.44750 (0.02)	1994.4 (42.12)	

**Table 6:** Findings of “Yalkowsky-Roseman model” for ITN (3) in several {DMSO (1) + H_2_O (2)} combinations at 298.2–318.2 K.

*m*	log *x*^Yal^					Overall *RMSD* (%)
	*T* = 298.2 K	*T* = 303.2 K	*T* = 308.2 K	*T* = 313.2 K	*T* = 318.2 K	
0.1	−5.96	−5.80	−5.61	−5.46	−5.34	
0.2	−5.42	−5.28	−5.10	−4.96	−4.85	
0.3	−4.87	−4.75	−4.59	−4.47	−4.37	
0.4	−4.33	−4.22	−4.08	−3.97	−3.89	2.10
0.5	−3.79	−3.69	−3.57	−3.48	−3.40	
0.6	−3.24	−3.16	−3.06	−2.98	−2.92	
0.7	−2.70	−2.63	−2.55	−2.49	−2.44	
0.8	−2.15	−2.10	−2.04	−1.99	−1.95	
0.9	−1.61	−1.57	−1.54	−1.50	−1.47	

**Table 7:** Findings of “Jouyban-Acree” and “Jouyban-Acree-van’t Hoff” models for ITN (3) in different {DMSO (1) + H_2_O (2)} mixtures (values in parentheses are standard deviations of model parameters).

System	Jouyban-Acree	Jouyban-Acree-van’t Hoff
		*A*_1_ 0.52410 (0.01) *B*_1_ −892.54 (28.41) *A*_2_ 10.402 (0.34) *B*_2_ −7568.3 (512.12) *J*_i_ 29,178 (532.41) 1.15
{DMSO (1) + H_2_O (2)}	*J*_i_ 30,624 (561.32)	
	
	
*RMSD* (%)	1.02	

**Table 8:** Apparent thermodynamic parameters (Δ_sol_*H*^0^, Δ_sol_*G*^0^, and Δ_sol_*S*^0^) along with *R*^2^ values for ITN (3) in different {DMSO (1) + H_2_O (2)} mixtures ^c^ (values in parentheses are standard deviations of thermodynamic parameters).

*m*	Δ_sol_*H*^0^/kJ mol^−1^	Δ_sol_*G*^0^/kJ mol^−1^	Δ_sol_*S*^0^/J mol^−1^ K^−1^	*R* ^2^
0.0	63.00 (0.76)	36.27 (0.43)	86.78 (1.12)	0.9966
0.1	57.15 (0.67)	33.20 (0.41)	77.75 (1.03)	0.9971
0.2	52.98 (0.63)	30.19 (0.39)	73.99 (1.00)	0.9967
0.3	46.19 (0.52)	27.15 (0.37)	61.83 (0.98)	0.9969
0.4	41.89 (0.48)	24.14 (0.36)	57.59 (0.92)	0.9968
0.5	35.40 (0.42)	21.13 (0.30)	46.31 (0.81)	0.9967
0.6	31.28 (0.40)	18.10 (0.26)	42.78 (0.74)	0.9940
0.7	23.95 (0.32)	15.10 (0.24)	28.74 (0.56)	0.9978
0.8	20.05 (0.28)	12.07 (0.20)	25.90 (0.48)	0.9989
0.9	13.35 (0.22)	9.078 (0.14)	13.87 (0.23)	0.9948
1.0	7.430 (0.10)	6.077 (0.09)	4.392 (0.10)	0.9992

^c^ The relative uncertainties are *u*(Δ_sol_*H*^0^) = 0.051, *u*(Δ_sol_*G*^0^) = 0.047 and *u*(Δ_sol_*S*^0^) = 0.057.

**Table 9:** Summary of materials used.

Material	Molecular Formula	Molar Mass (g mol^−1^)	CAS RN	Purification Method	Mass Fraction Purity	Analysis Method	Source
ITN	C_20_H_28_O_2_	300.40	4759-48-2	None	>0.98	HPLC	BOC Sciences
DMSO	C_2_H_6_OS	78.13	67-68-5	None	>0.99	GC	Sigma Aldrich
Water	H_2_O	18.07	7732-18-5	None	-	-	Milli-Q

ITN: isotretinoin; DMSO: dimethyl sulfoxide; HPLC: high-performance liquid chromatography; GC: gas chromatography.

## Data Availability

The data are available on reasonable request from the corresponding author.

## Sample Availability

Samples of the compound ITN are available from the authors.
